# Supplementary material for: Erythema Increase Predicts Psoriasis Improvement after Phototherapy
Source: J Clin Med. 2021 Aug 30;10(17):3897. doi: 10.3390/jcm10173897 (PMC8432224; doi:10.3390/jcm10173897)
Supplement: Supplementary file 1 [file jcm-10-03897-s001.zip › jcm-1322178-supplementary.pdf]

Supplementary Material

Table S1. Characteristics of the participants included in the study.

	All participants at baseline (*n* = 76)	Non-exposed participants at baseline (*n* = 38)	Exposed participants at baseline (*n* = 38)	All participants with follow-up (*n* = 52)	Non-exposed participants with follow-up (*n* = 26)	Exposed participants with follow-up (*n* = 26)	p*	p**
**Age (years)**	43.03 (17.48)	44.92 (17.17)	45.13 (18.17)	44.77 (17.15)	45.00 (16.51)	44.54 (18.08)	0.959	0.85
**Sex (%)**								
**-Female**	40 (52.6%)	20 (52.60%)	20 (52.6%)	24 (46.2%)	12 (46.2%)	12 (46.2%)	1.00	1.00
**-Male**	36 (47.4%)	18 (47.40%)	18 (47.4%)	28 (53.8%)	14 (53.8%)	14 (53.8%)		
**Phototype**								
**- II**	6 (7.9%)	2 (5.3%)	4 (10.50%)					
**-III**	66 (78.9%)	33 (86.8%)	30 (78.90%)				0.781	0.621
**-IV**	7 (9.2%)	3 (7.9%)	4 (10.50%)					
**Smoking habit**								
**- Non-smoker**	62 (81.6%)	32 (84.20%)	30 (78.9%)	45 (86.5%)	23 (88.5%)	22 (84.6%)	0.554	1.00
**- Smoker**	14 (18.4%)	6 (15.80%)	8 (21.1%)	7 (13.5%)	3 (11.5%)	4 (15.3%)		
**Alcohol habit (yes)**	35 (46.1%)	17(44.70%)	18 (47.4%)	26 (46.2%)	12 (46.2%)	12 (46.2%)	0.818	1.00
**Family history of psoriasis (yes)**	18 (28.9%)	0 (0.00%)	18 (47.4%)	11 (21.2%)	0 (0.0%)	11 (42.3%)	<0.001*	<0.001**
**Emollients use (yes)**	41 (53.9%)	16 (42.10%)	25 (65.8%)	26 (50.0%)	9 (34.6%)	17 (65.4%)	0.038*	0.027**
**DLQI**								
**- Baseline**			7.91 (6.61)			6.92 (5.71)	-	-
**- After 15 phototherapy sessions**			-			4.88 (5.41)		
**PASI**								
**- Baseline**			8.55 (4.34)			7.86 (4.44)	-	-
**- After 15 phototherapy sessions**			-			4.72 (4.00)		
**BSA**								
**- Baseline**			11.02 (8.54)			10.19 (8.60)	-	-
**- After 15 phototherapy sessions**			-			6.35 (5.12)		
**Disease duration (years)**			13.59 (11.46)			15.42 (11.22)	-	-
**Previous treatments**								
**- Topical corticosteroids**			28 (100.00%)			26 (100.00%)	-	-
**- Systemic drugs**			7 (18.40%)			6 (23.10%)		
**- Biologic drugs**			3 (7.89%)			3 (11.5%)		
**Session dose (Joules)**								
**- Baseline**			0.42 (0.27)			0.46 (0.31)	-	-
**- After 15 phototherapy sessions**			-			1.41 (0.16)		
**Session time (seconds)**								
**- Baseline**			114.84 (82.00)			127.48 (96.95)	-	-
**- 15th phototherapy sessions**			-			366.62 (50.22)		

BSA, Body Surface Area; DLQI, Dermatology Life Quality Index; NB-UVB, Narrow-Band Ultraviolet B; PASI, Psoriasis Area and Severity Index.Data are expressed as relative (absolute) frequencies and means (standard deviation (SD). The Student's t test for independent samples or Welch's test, as appropriate, were used to compare continuous variables and the chi-square test or Fisher's exact test, as appropriate, were applied to compare categoric data. Two-tailed $p < 0.05$ was considered statistically significant in all tests. **p* value to compare non-exposed participant and exposed participants at baseline after using Student's t test for independent samples or Welch's test, as appropriate, to compare continuous variables; and the chi-square test or Fisher's exact test, as appropriate, to compare categoric data. ***p* value to compare non-exposed participant and exposed participants with follow-up after using Student's t test for independent samples or Welch's test, as appropriate, to compare continuous variables; and the chi-square test or Fisher's exact test, as appropriate, to compare categoric data.

Table S2. Sensitivity and specificity in the prediction of clinical improvement after 15 phototherapy session based on the skin homeostasis changes after one phototherapy session.

	Cutoff value	Sensitivity	Specificity	OR	p
Erythema (AU)	**53.23**	**71.4**	**84.2**	**13.33**	**0.013***
SCH (AU)	**1.06**	**71.4**	**63.8**	**4.29**	**0.130**
2 criteria (erythema>53.23 + SCH>1.06)	**-**	**57.1**	**94.7**	**24.00**	**0.013***

AU, arbitrary units; OR, odd ratio; SCH, Stratum Corneum Hydration;
